# Leukocyte Coping Capacity: An Integrative Parameter for Wildlife Welfare Within Conservation Interventions

**DOI:** 10.3389/fvets.2019.00105

**Published:** 2019-04-11

**Authors:** Nikolaus Huber, Valeria Marasco, Johanna Painer, Sebastian G. Vetter, Frank Göritz, Petra Kaczensky, Chris Walzer

**Affiliations:** ^1^Department of Integrative Biology and Evolution, Research Institute of Wildlife Ecology, University of Veterinary Medicine Vienna, Vienna, Austria; ^2^Department of Integrative Biology and Evolution, Konrad Lorenz Institute of Ethology, University of Veterinary Medicine Vienna, Vienna, Austria; ^3^Institute of Biodiversity, Animal Health and Comparative Medicine, University of Glasgow, Glasgow, United Kingdom; ^4^Department of Reproduction Management, Leibniz Institute for Zoo and Wildlife Research, Berlin, Germany; ^5^Department of Terrestrial Ecology, Norwegian Institute for Nature Research, Trondheim, Norway; ^6^Wildlife Conservation Society, Bronx, NY, United States

**Keywords:** stress, leukocyte coping capacity, endocrine-immune interaction, animal welfare, wildlife management, conservation interventions

## Abstract

Wildlife management, conservation interventions and wildlife research programs often involve capture, manipulation and transport of wild animals. Widespread empirical evidence across various vertebrate taxa shows that handling wildlife generally induces a severe stress response resulting in increased stress levels. The inability of individuals to appropriately respond to rapidly changing environmental conditions during and after manipulations may have deleterious and long-lasting implications on animal welfare. Therefore, mitigating stress responses in the frame of conservation interventions is a key animal welfare factor. However, we have a poor understanding of the metrics to adequately assess and monitor the dynamic physiological changes that animals undergo when subjected to stressful procedures in wild or captive conditions. A growing number of studies provide good evidence for reciprocal interactions between immune processes and stress. Here, we review the existing literature on a relatively new technique—Leukocyte Coping Capacity (LCC), a proxy for stress quantifying oxygen radical production by leukocytes. We discuss the strength and weaknesses of this immunological approach to evaluate stress, the individual capacity to cope with stress and the resulting potential implications for animal welfare. Additionally we present new data on LCC in captive roe deer (*Capreolus capreolus*) under long-time anesthesia and free-ranging Asiatic wild asses (Kulan; *Equus hemionus kulan*) were LCC was used to assess stress levels in animals captured for a reintroduction project.

## Stress and Animal Welfare

With increasing human impact on natural ecosystems, the need for “hands-on” wildlife conservation and management is on the rise [e.g., (1–3)]. Conservation interventions frequently require capture, manipulation and transport of individuals, but the concomitant and potential long-lasting effects on the target animals are often overlooked (4–7). Only few studies have investigated the impacts of conservation activities on wildlife health and welfare (8–10).

The broad definition of “Animal welfare” involves the well-being of animals based on the underlying psychological and physiological ability of the individual to cope with changes in its immediate environment (11–13). Difficulties or the inability to cope with environmental pressures can lead to stress and hence potential negative impacts on animal health and well-being as well as decreased resilience (14–16). Moberg (17) proposed that determining to which extent an animal is impacted from stress due to changes in its biological functions, thereby entering a pre-pathological state, is the only defensible measurement of well-being in animals (17, 18). Accordingly, the definition of potential stressors and the further development of methods to measure and assess stress responses are crucial for the evaluation of wildlife welfare (19–21).

The term “stress” is a notoriously ambiguous concept in biology and medicine. After the earlier definitions of the term by Cannon (22) and Selye (23) which were broadly based on the “non-specific responses of the body to any demand for change” [see (24), for a comprehensive review on the definition of stress] Sterling and Eyer (25) and later (16, 26) introduced the concept of “Allostasis.” This concept can be summarized as the process of “achieving stability of the internal milieu (homeostasis) through change.” This definition accounts for daily and circannual physiological adjustments that constantly occur during the life cycles of animals. More recently the allostasis concept was extended within the reactive scope model, which integrates the importance of species developmental strategies and their potential long-lasting impact in priming and programming later life stress responses (24).

Beyond the mere definition of stress, which due to the complexity and multi-dimensionality of the phenomenon may be hard to frame, the main physiological systems for coping with stressors are relatively well-studied. There are two major mediators orchestrating the stress response in vertebrates: (i) catecholamine's controlled by the sympathetic nervous system (SNS) and (ii) glucocorticoid stress hormones [GCs; corticosterone in amphibians, reptiles and birds, cortisol in most fish and mammals—(27)] modulated by the Hypothalamic-Pituitary-Adrenal axis (HPA-axis). Activation of the SNS triggers the release of catecholamine's within milliseconds after the onset of a stressor for immediate responses such as the “fight or flight” response [Cannon (22), recently reviewed by Romero and Wingfield (28)]. The HPA axis response is slower (within minutes) and acts on various physiological pathways to adjust essential bio-regulatory mechanisms in response to stressors, such as extreme weather conditions, predator exposure, or shortages of food (15, 29). This is primarily achieved by up-regulating key body functions, including cardiac-, respiratory- and brain-activity as well as energy mobilization at the expense of other processes such as growth, reproduction, immunity or the balance between oxygen radicals and the antioxidant system (29–31).

In general shorter-term/acute stress responses are thought to have an adaptive fitness value, whereas longer-term/chronic exposure to stress are generally associated with persistent immune modulation and an increase in susceptibility to diseases (17, 26, 29, 32). However, nature, duration and magnitude of stressful events are likely to be fundamental in determining the biological benefits or costs of exposure to stress (29, 33). There is growing evidence suggesting that the long-term and repeated exposure to moderately challenging stressors is associated with positive, rather than negative, organismal outcomes, improving survival and delaying the onset of reproductive senescence (34, 35). It is therefore key to assess and quantify how and to which extent differing stressors such as those provoked during wildlife and conservation management activities (i.e., capture, handling, transport, relocation) impact on individual responses and consequently on animal welfare (36, 37).

## Measuring Stress

Stress responses vary vastly among species as well as within individuals of the same species (28, 38–40), are modulated by season, time of day (41) and can be triggered by a great variety of stressors (42). Moreover, stress responses involve several physiological processes in parallel and are therefore difficult to measure and to assess, particularly with the small sample sizes typical in field studies of wildlife species (43). Currently physiological stress responses in wildlife are assessed with a variety of techniques (20) including measuring GCs in various tissues (44–46), changes in blood chemistry and hematology (47) and behavioral alterations, such as exploratory or avoidance behaviors (48). Measuring GCs has generally been adopted as a standard procedure to estimate individual stress levels. However an elevation of GCs does not necessarily always indicate a state of stress or discomfort, as baseline and stress GCs levels can fluctuate hugely among an individual's life history stages (49, 50). Therefore, the use of GCs as a single metric to gain a comprehensive understanding of individual stress conditions is limited (50). While there has been an over-reliance upon GCs, other pathways of the stress response, such as endocrine-immune interactions as proxies for stress and animal welfare, are surprisingly understudied. In order to better understand the causalities and complex mechanisms within the stress response and its implications for animal welfare, it is imperative to integrate different approaches to better assess and interpret the phenomenon of stress (43, 51).

## Immune Markers As a Potential Proxy For Stress And Animal Welfare

Several studies provide solid evidence for the strong and reciprocal interaction between immune processes and stress (52–54). It is now widely accepted that the immune system and the neuroendocrine system form an integrated and evolutionary highly conserved element of physiology across phyla (55, 56). Therefore, direct and indirect stress-induced effects on quantitative and functional immune parameters can serve as additional markers to assess stress and wildlife welfare. The best established and most commonly used immune parameter applied across all five vertebrate taxa is the stress-related change in immune cell distribution (i.e., leukocyte profiles). Higher stress levels are associated with an increase of neutrophil granulocytes (heterophils in bird and reptile species) and a decreae of lymphocytes in the bloodstream and hence an increase in neutrophil to lymphocyte ratio [N:L; (57)] [for review see (47, 58)]. Singh (59) lists acute phase response protein levels, natural serum antibody levels, the phagocytic capacity of Natural Killer cells, γδ- receptor positive T-lymphocytes, and stress-induced changes in inflammatory cytocine levels (interleukines and tumor necrosis factors) as innate immune markers which can be used to infer welfare outcomes. Another interesting immunological marker for stress is neopterin, a pteridine derivate synthetized by monocytes and macrophages upon inflammatory cytokine stimulation. Serum neopterin levels in pigs significantly increased after a 30 min transport phase and could be a useful marker to quantify acute/short-term stress-induced cellular immune stimulation (60). Another promising immune marker appears to be Immunoglobulin A (IgA) and in particular its secretory form (SIgA) the major antibody of mucosal immune defense in mamals and birds. The review by Staley et al. (21) reports that long-term examinations of IgA levels reveal consistent patterns with a suppression of SIgA after periods of psychological or physical chronic stress. In contrast, situations with good or enhanced welfare, lead to increased SIgA levels suggesting that this marker can be a suitable immunological proxy for animal welfare.

## Leukocyte Coping Capacity as a Proxy For Stress

Polymorphonuclear leukocytes (PMNLs), i.e., primarily neutrophil granulocytes in mammals (61) and heterophil granulocytes in birds (62), are the first line of innate immune protection in vertebrates. They become activated when binding to surface peptides of pathogens or by the stress-related activation of their α- and β-adreno- (63, 64) and glucocorticoid receptors (65). Once activated, PMNLs perform the so called ‘oxidative burst' and produce superoxide free radicals as the basis for a suite of anti-pathogenic reactive oxygen species (ROS) generated upon the NADPH oxidase enzyme complex (66). An example emphasizing the biological significance of this innate immune reaction is chronic granulomatous disease (CGD), an inherited immunodeficiency in humans, where PMNLs are not able to generate ROS upon stimulation. CGD and an insufficient oxidative burst response in general, are characterized by recurrent bacterial and fungal infections and a set of inflammatory complications with not uncommonly, lethal outcome (67, 68).

In wildlife the initial stress-induced oxidative burst of PMNLs acts as immediate protection against invading pathogens in the case of injury by a predator (69, 70). However, the capability of PMNLs to produce further ROS after the initial (stress induced) burst is curtailed to protect the organism from over-activation of PMNLs while reducing free radical damage of surrounding tissues (71, 72). Therefore, during short-term stress PMNL ROS production denotes an immediate stress response which is rapidly curtailed (71, 72). On the other hand, if stress conditions persist, this innate immune response is diminished to depleted with detrimental impacts for the health, welfare and survival of the individual (70, 73–75).

McLaren et al. (76) developed a method called Leukocyte Coping Capacity (LCC), using PMNLs and the change in their reactivity as bio-indicators for measuring stress events (76). PMNLs have over 150 different receptors which are sensitive to varied stress signals in the organism, including plasma endocrine factors, changes in blood biochemistry and red cell hemodynamics, changes of cytokine levels and mediators released by the HPA axis and the SNS (72). This synchronous sensitivity to several stress mediators and an array of stress- related physiological changes emphasizes PMNLs as excellent indicators in evaluating stress levels (77). The technique relies on the observation that PMNLs of stressed individuals have a reduced capacity to produce ROS in response to a secondary (chemical) external stimulus (78). Thus, low LCC levels in an individual indicate a decreased innate immune response and increased stress levels.

Despite the sensitivity of PMNLs to an array of constituent mediators of the stress response, the physiological relevance of the method is promising for the following reasons: (i) PMNLs remain in their natural environment, i.e., in whole blood, allowing dynamic, and three dimensional interactions with other surrounding blood cells (e.g., macrophages or erythrocytes) as well as cell–cell interactions within and among different leukocyte cohorts, (ii) the method does not necessitate centrifugation known to change cell reactivity and also avoids “plating out” cells on glass slides as in other approaches to determine PMNL activation [Nitro blue tetrazolium test—Montes et al. (79)], minimizing the disruption of important cell signaling pathways and maintaining PMNL responsiveness and integrity, (iii) the response can be followed in real-time via direct quantitative chemiluminescence readings (80), (iv) the interaction between the immune- and stress systems is evolutionary highly conserved and therefore the LCC technique can be applied potentially across all wildlife species (78, 81, 82). For further information on details of the LCC protocol see Supplementary Material S1.

The method provides several additional technical advantages: (i) a relatively small amount of blood (i.e., 20 μl) is needed to perform the assay, making it applicable for small vertebrate species, e.g., rodents, passerine bird or bat species; (ii) the procedure is rather simple, minimizing sources for error, and (iii) the response can be measured via a portable Chemiluminometer (e.g., Junior LB 9509, EG & G Berthold, Germany) providing immediate results, which is a great advantage in field studies in free-living animals.

### Confounding Factors and Constrains

Measuring stress with the LCC protocol is still relatively novel. There are several aspects which require further experimental testing to establish the diagnostic efficacy of the methodology. It should be noted that studies investigating the relationship of LCC to more commonly used proxies for stress (e.g., heart rate, N:L ratio, blood glucose or circulating cortisol levels) did not find correlative relationships (77, 83, 84). This lack of correlation may be explained by large individual variation in stress responses as well as by differing physiological strategies to cope with stress and/or the diverging operative time frames of pathways and mediators involved into the stress cascade (39, 40, 43). An additional explanation may be the synchronous sensitivity of PMNLs to several stress related changes (77). During infection and disease a multitude of immunological factors are altered. Neutrophil “priming” agents such as chemoattractants (e.g., bacterial peptides/proteins), inflammatory cytokines (e.g., tumor necrosis factor alpha) or Toll-like receptor agonists (e.g., endotoxins) all have the potential to increase PMNL ROS production (72, 85) and potentially bias LCC dynamics. Gonadal steroids (e.g., androgens and estrogens) may have direct effects on the ROS production of PMNLs and alter LCC responses during times of reproduction, although previous studies on this topic provided contrasting results (86–88). Future studies will need to assess stress hormone, and gonadal steroid effects on the LCC response in order to better elucidate functional endocrine-immune interactions that could be linked with animal welfare. We also need further studies to elucidate the down-stream mechanisms triggering PMNL activation and relevant time windows in which these pathways do operate. However, there are no clear physiological profiles of ensured welfare within a species or even between individuals. Hence future studies should aim for a systematic, multivariate approach including several parameters of physiological and behavioral nature to gain more insight toward the validity of potential tools such as LCC to assess stress and welfare (89–92).

Capture and handling of wildlife species often involve anesthesia of individuals with varying protocols which are constantly adapted for animal safety and welfare reasons (93, 94). Anesthetic agents have the potential to decrease PMNL oxidative burst capacity in humans. This decrease has been shown for opioids (morphine), thiopental, propofol, midazolam, volatile anesthetics (i.e., halothane, isoflurane, and sevoflurane) and local anesthetics (lidocaine, bupivacaine). In contrast ketamine and synthetic opioids (fentanyl, remifentanil, and alfentanyl) did not alter PMNL ROS production (95, 96); for review see Kurosawa and Kato (97). However, despite some studies in humans [e.g., (98, 99)] and one in a fish species (100), studies on the effect of anesthetic agents on PMNL function in wildlife are to date lacking.

### Studies Inferring LCC as a Valid Proxy to Assess Stress in the Context of Welfare

A review on phagocyte photon emission in response to stress and disease noted that the capability of PMNLs to emit ROS reflects the pathophysiological state of the host and that the magnitudes of stress as well as the presence of pathogens and disease processes can be estimated (81). A later study in Atlantic salmon (*Salmo salar*) revealed that fish subjected to a 2 h period of confinement stress had a reduction in oxygen free radical production in isolated PMNLs and therefore a lower oxidative burst capacity and a debilitated innate immune response (78). In Table 1, we review a sample of studies inferring LCC as a valid proxy to assess stress and animal welfare. McLaren et al. (76) used the LCC method to examine the effects of transport from a capture site to a field laboratory in wild badgers (*Meles meles*). The study showed that transported individuals (ca. 10 min on a trailer pulled by an all-terrain quad) exhibited a detectable reduction in LCC levels when compared to individuals sampled directly at the capture site. These data indicate that transport is likely to be a compounding stressor beyond the capture event (76). A study on bank voles (*Clethrionomys glareolus*) and wood mice (*Apodemus sylvaticus*) indicates that handling *per se* is likely to alter LCC responses. Handled animals (only for 20 s) showed remarkable reduction in LCC in comparison to non-handled animals (102). In non-anesthetized European Roe deer (*Capreolus capreolus*) LCC levels were negatively impacted by the time of human presence at the capture site prior to the actual handling procedure, suggesting that human presence at the trapping site prior to handling should be minimized (84). The LCC technique was used to investigate the stress response caused by capture and subsequent abdominal surgery of free-ranging brown bears (*Ursus arctos*) and to evaluate whether variation in LCC co-varied with other proxies of metabolic and physiological stress, such as heart rate, N:L—ratio, blood glucose and circulating cortisol concentrations (83). Their main result revealed that LCC values following capture were lower in solitary bears when compared to females with cubs and lower in bears in poorer body condition when compared to those in good body condition. LCC levels did not seem to be influenced by the actual surgical procedure under anesthesia (83). A recent study comparing blood glucocorticoid levels, hematology, LCC, scrotal, and perineal temperature, scrotal lesion, and a pain score in two groups of male calves (*Bos Taurus*), a ring castration and a sham castration control group, suggests LCC as an innovative tool for stress and pain assessment (105).

**Table 1:** Overview of studies inferring LCC as a valid proxy to assess stress and welfare in animals.

**Species**	**Context**	**Change in LCC**	**Remarks**	**References**
Badger (*Meles meles*)	Capture, transport, handling	↓ Transport	Transport was identified as additional stressor prior to handling	(76)
Scandinavian brown bear (*Ursus arctos*)	Capture via helicopter, surgery	↓ Capture	Variation in LCC was best explained by social status	(83)
		↑ During anesthesia	Bears in better body condition coped better with capture and handling	
Water vole (*Arvicola terrestris*)	Captive housing, social stress	↓ Group size	Individuals held in large groups showed greater declines in LCC	(101)
Bank vole (*Clethrionomys glareolus*)	Trapping and short handling	↓ Handling	Even a short period of 20 s of handling induces a decrease in LCC	(102)
Wood mice (*Apodemus sylvatikus*)			Note: potential bias by the use of isoflurane during handling	
Water vole (*Arvicola terrestris*)	Captive conditions, handling,	↓ Captivity	Indoor-housing caused a greater decline in LCC compared to outdoor- conditions	(103)
	Radio collaring	↓ Indoor housing	Continuous decrease of LCC over the entire experiment (6 weeks)	
		↓ Collaring	LCC of collared individuals decreased more within the first week of the exp.	
European roe deer (*Capreolus capreolus*)	Capture and handling	↓ Prior to handling	LCC levels were negatively correlated with the time of human presence prior to the handling procedure prior to the handling	(84)
House sparrow (*Passer domesticus*)	Capture and handling	↓ Capture, handling	Capture induced a decrease in LCC	(51)
		↑ During confinement	LCC of birds kept in a cotton bag recovered during a 30 min period	
		↓ Females	Females showed significantly lower LCC levels in response to the stressor	
Rhesus macaques (*Macaca mulatta*)	Captive conditions	↓ Caged housing	Caging system caused significantly lower LCC responses compared with open rooms	(104)
Kulan (*Equus hemionus*)	Capture for reintroduction	↓ In agitated indiv.	Suggests LCC has the potential to identify high risk candidates	Huber et al. this study
European Roe deer (*Capreolus capreolus*)	Long-term anesthesia monitoring	↑ Until 80 min and↓ thereafter	Suggests LCC as a useful tool for anesthesia monitoring	Huber et al. this study
		↓ In winter	Marked seasonal difference in LCC with lower levels in winter	
Cattle (*Bos taurus*)	Ring castration	↓ Ring castration	Lower LCC in ring castrated calves during the degenerative phase of scrotal tissue	(105)

Within a reintroduction program for conservation purposes Moorhouse et al. (103) analyzed the impact of housing conditions, handling procedures and radio-collaring in captive bred water voles (*Arvicola terrestris*) via LCC measurements. The authors found a larger decrease in LCC levels between week 1 and 2 for individuals that were radio-collared while this was not the case in non-collared individuals, suggesting that radio-collaring could be an additional stressor, at least in this species. In this experiment one group of individuals were housed in outdoor enclosures and the other group in indoor laboratory cages. LCC values of both groups decreased constantly over the 6-week study period, but interestingly, animals housed indoors and individually in laboratory cages showed lower LCC values despite the fact that they usually do not live in large groups and are territorial in the wild (103). This result partially contrasts results from (101), who examined short-term social stress by means of body weight change and LCC to test the effects of group size in captive-bred water voles destined for release within a reintroduction program. LCC scores were negatively correlated with group size, suggesting that individuals held in larger groups experienced higher relative levels of stress and therefore showed a greater decline in LCC (101). Moorhouse et al. (103) interpreted the overall continuous decrease in LCC values as the cumulative result of repeated-handling induced stress. The latter study and Gelling et al. (101) also suggest that the preferred social structure needs to be considered in order to reduce stress levels and enhance wildlife welfare within conservation projects. Honess et al. (104) likewise applied the LCC method (referred to as “neutrophil activation test”) to assess differences in stress levels between different housing conditions in a breeding colony of rhesus macaques (*Macaca mulatta*). Individuals were housed either in a caging system (reinforced stainless steel two-tier laboratory cages) or open-rooms. Animals in the caging system exhibited significantly lower LCC responses when compared to animals held in open rooms, indicating that cage housing is associated with diminished immune function as well as higher stress levels and therefore impaired welfare (104). The LCC method was recently tested in an avian species, the house sparrow (51). It was shown that after an initial decrease LCC levels increased during a 30 min time period after the captive birds where confronted with the acute stressor of a standardized capture and handling (106). LCC levels during the acute stress response were compared to circulating concentrations of GCs (i.e., corticosterone) and markers of oxidative stress in two different seasons, winter and spring, respectively. All three methodologies detected significant changes due to the acute stressor but they were not correlated with each other. There were marked seasonal differences in GC response, with higher levels in spring in both sexes. Had the study measured the classical approach of measuring total GCs, the most obvious conclusion would have been that individuals confronted with the same stressor experienced a higher short-term stress response in spring when compared to winter, with no difference between sexes. On the other hand, simultaneous LCC measures revealed similar stress responses during both seasons with marked sex differences in relative stress levels and thus in the ability to cope with the stressor. There was no change in oxidative stress levels at the expense of a decrease in anti-oxidative capacity (measured as the ability of serum to neutralize hypochlorous acid) 30 min after the acute stress event. Combining the three methodologies allowed, to some extent, for a more holistic appreciation of the stress response: the elevation of GC levels and the neutralizing effect of antioxidants on ROS in the circulation facilitated the reestablishment of homeostasis in the organism (Allostasis). This recovery was illustrated by the increase in LCC within a 30 min time period and reflects the restoration of the capacity to cope with repeated or novel stress (76). Results from this study clearly highlight the necessity of increasing the scope and number of physiological systems within the stress-endocrine-immune interface which need to be investigated concurrently in future studies to better assess and understand the complexity of coping mechanisms related to stress and the impacts on welfare.

In our perspective the above mentioned literature suggests LCC as a useful tool within wildlife management or conservation interventions (i.e., capture, handling, and transport, housing conditions). In order to further assess the validity of LCC to identify stress eliciting factors future studies should incorporate different intensities of identified or suspected stressors in a systematic approach (e.g., short- vs. long-human presence prior to handling) and include LCC in addition to other measures of stress and welfare (e.g., hormone levels, SIgA, behavioral scores). Such studies would be very important in order to optimize exposure to the tested stressors, thereby increasing animal welfare and furthering our understanding of the extent to which different stressors alter LCC responses.

### Latest LCC Data From Two Ongoing Wildlife Projects

**Figure 1:**
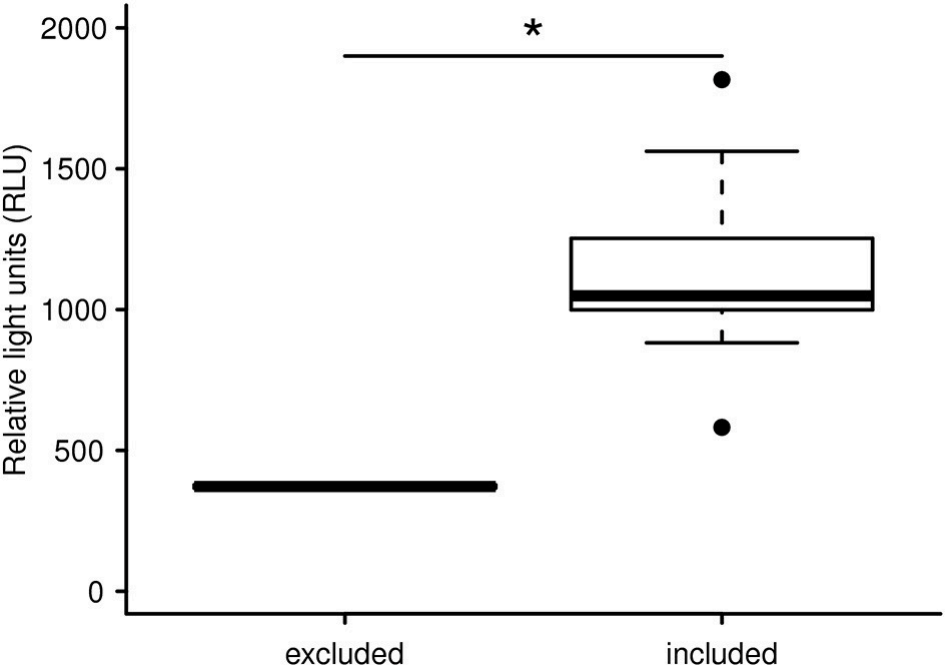
LCC peak levels (expressed in relative light units) of free ranging Asiatic wild ass after capture and handling within a reintroduction project in Kazakhstan. The LCC of two overly agitated individuals are significantly lower compared to 10 animals not showing signs of increased agitation. The two severely stressed kulan were excluded from the transport and translocation for animal welfare reasons. The asterisk indicates a significant difference of *p = 0.013.

**Figure 2:**
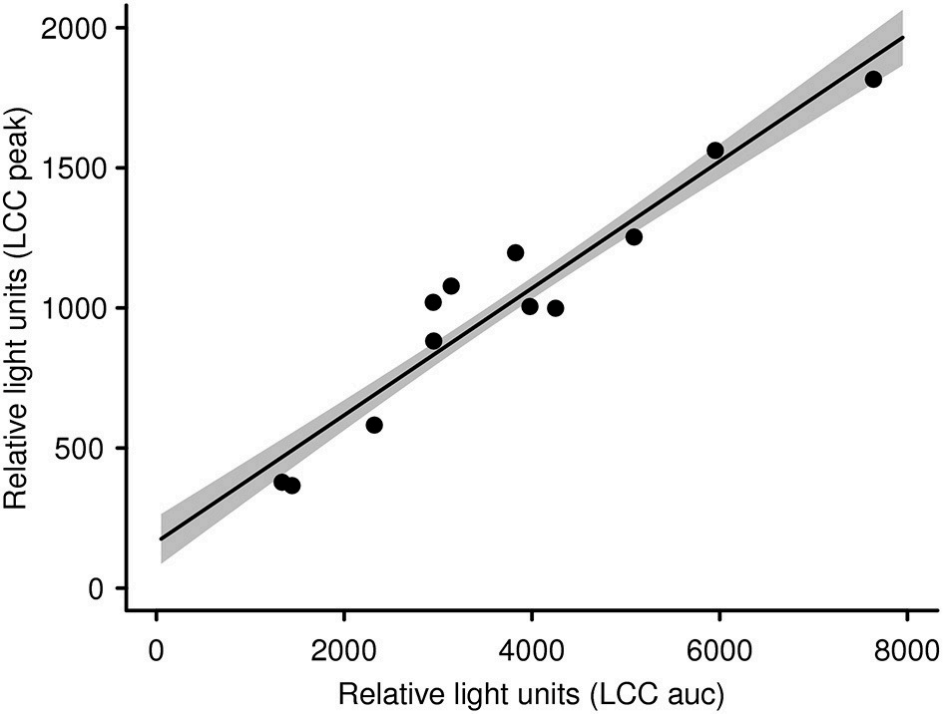
LCC peak levels in kulan are significantly linked with and therefore representative for the whole LCC curve (i.e., area under the curve; see also Figure S1). The gray shaded area represents the standard error of the slope.

With the aim of evaluating capture and handling procedures and to further expand the LCC approach to different vertebrate species, we measured LCC in 12 kulan (*Equus hemionus*) captured in Kazakhstan during a translocation project. In brief, kulan had been driven into a capture corral, rested overnight, then anesthetized via remote darting and subsequently sampled, radio-collared, and boxed for translocation the following day (107). Two kulan out of 12 had to be released from the transport boxes prematurely due to severe stress and danger from self-inflicted injury. By comparing the LCC peak values of these 2 prematurely released individuals vs. the 10 transported kulan, we were able to identify a significant difference between the two groups (Figure 1). This finding suggests that LCC measures on-site in the field may be a powerful animal welfare tool allowing the identification of overly excited individuals (potentially severely stressed), which have an increased risk of injury and mortality. Especially in situations where a subset of animals is selected for further handling or transport, LCC data might guide (i) the selection of the least stressed individuals, (ii) the exclusion of the most stressed individuals, and (iii) in expediting appropriate interventions for those individuals which most likely have an insufficient ability to cope with capture and handling. This study also confirmed findings from a previous study in roe deer (*Capreolus capreolus*) in which LCC peak values were shown to be a robust proxy for the entire LCC curve [Figure 2; (84)].

**Figure 3:**
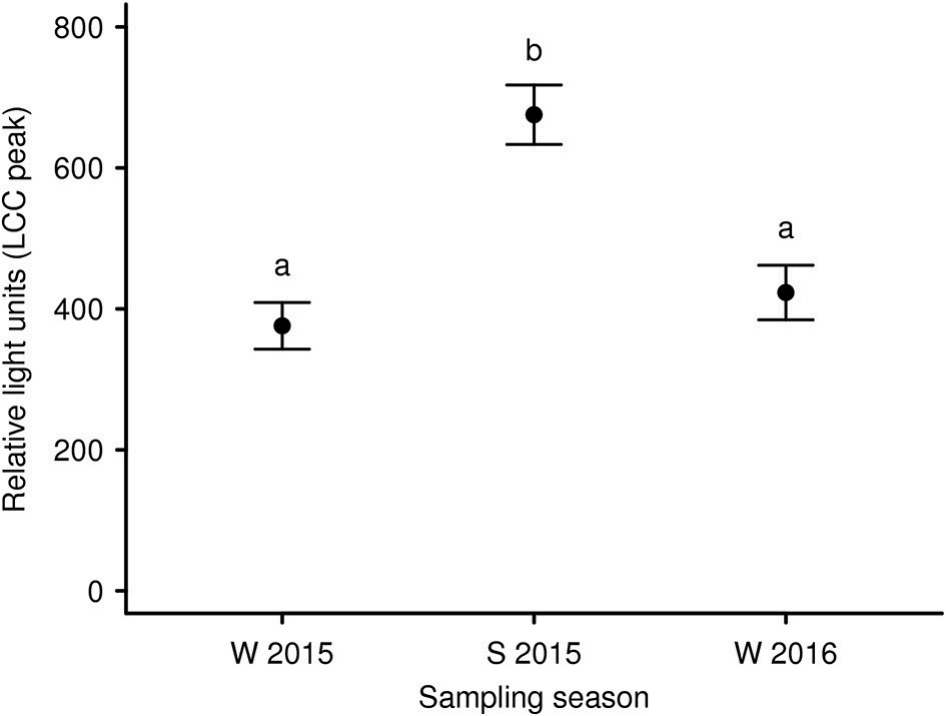
Mean LCC levels (± s.e.m.) of 9 European roe deer males during a 120 min period of anesthesia and split by seasons (W 2015: winter 2015; S 2015: summer 2015, and W 2016: winter 2016). Blood samples were taken as soon as the animals were in lateral or sternal recumbency due to anesthesia (T0) as well as 40 min (T40), 80 min (T80), and 120 min (T120) thereafter. Throughout all seasons the same 9 individuals were sampled. Different letters indicate significant *post-hoc* pairwise contrasts (*p* < 0.05 after Tukey's multiple comparison adjustment).

**Figure 4:**
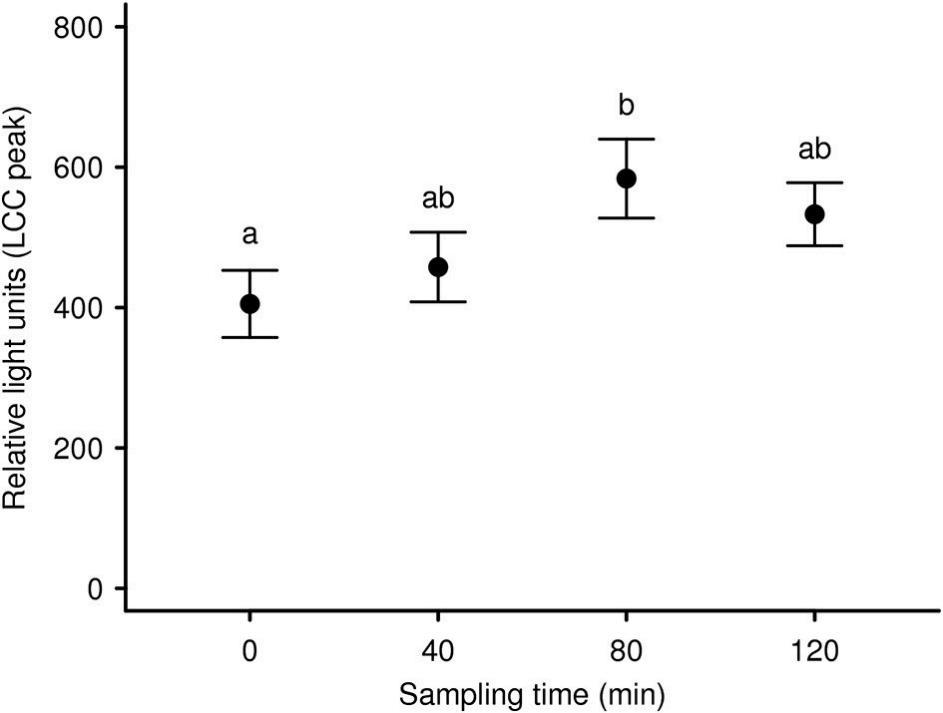
Mean LCC levels (± s.e.m.) of 9 European roe deer males during a 120 min period of anesthesia separated by sampling/bleeding time. The first sample was taken as soon as the animals were in lateral or sternal recumbency due to anesthesia (T0) as well as 40 min (T40), 80 min (T80), and 120 min (T120) thereafter. Throughout all seasons the same 9 individuals were sampled. Different letters indicate significant *post-hoc* pairwise contrasts (*p* < 0.05 after Tukey's multiple comparison adjustment).

To expand our knowledge concerning LCC dynamics and stress during long-term anesthesia (over a 120 min period) we analyzed data from 9 anesthetized captive European roe deer males. It was our aim to test recovery from initial capture and handling-induced stress (i.e., an increase in LCC) during the subsequent anesthesia, as observed in anesthetized brown bears (83). We found that season and sampling time significantly affected LCC levels in roe deer independently. LCC values during summer were markedly higher compared to two winter seasons (Figure 3). Supporting the work by Martin (32) these results suggest possible seasonality effects on the immune system. This highlights that seasonal impacts on the general capacity to cope with stressors as well as the cost to the immune system must be considered in study design. Ideally future studies will avoid or at least minimize capture and handling of roe deer during the winter in order to reduce stress levels and thereby improve welfare outcomes. We further identified a significant increase of LCC with increasing sampling time (i.e. the progression of anesthesia) suggesting a gradual recovery of innate immune function and capture stress during anesthesia (Figure 4). However, subsequently LCC values decreased non-significantly in all animals from T80 to T120. This result provides some evidence that LCC may be a useful tool for anesthesia monitoring detecting a possible threshold (a decrease after an initial increase in LCC levels) for ending the anesthesia to prevent the onset of cumulative negative impacts.

For full details on the two projects described here, including the LCC protocol, statistical analyses and results see Supplementary Materials S1, S2.

## Conclusion

There are several approaches such as shifts in hormone concentrations, blood parameters and behavior to assess stress and its implications for wildlife welfare (20). However, these common measures generally do not always provide robust and reproducible results, largely due to the challenges associated with the complexity of the neuro-endocrine systems (92). Moberg (18) stated that the biological cost of mounting a stress response is the key to determine the welfare implications of potential stressors and therefore would be more relevant when compared to other measures of stress such as physiological or behavioral changes (17, 18). The LCC technique provides a window to assess the biological costs associated with the impaired capacity of PMNLs to mount an oxidative burst after a stressful event. A reduction of LCC directly reflects increased stress levels and reduced (innate) immune function. This denotes a “pre-pathological” state which engenders costs, may be predictive for a breakdown in biological functions and is subsequently a promising indicator of animal well-being (76, 83, 84, 108). Due to the fact that LCC captures some of the complexity of action and reaction of PMNLs to a multitude of stress signals within and among animal species and their environment this method provides holistic insights into the trade-off and associated costs between stress response and immune function. However, a combined approach using two or ideally more stress parameters provides a far more comprehensive approach when evaluating stress and animal welfare impacts.

Our review suggests that measuring LCC has the potential, amongst others, to develop in the short-term into a helpful tool to disentangle the stressful components of capture, trapping and handling procedures in wildlife. Given the implications that animal welfare perception has on the acceptance of wildlife conservation and management interventions, information provided by new techniques, such as LCC, will allow researchers to better evaluate and communicate the impact of their work while adjusting and refining procedures and protocols accordingly.

## Ethics Statement

This study on the European roe deer was carried out in accordance with the recommendations of the German Ministry for Environment, Health and consumer protection and all experimental procedures were approved by the ethical committee of the German Ministry for Environment, Health and consumer protection (AZ: 2347-4-2015). The Kulan project (ecological assessment) was approved by the Committee of Forestry and Wildlife (CFW) of the Ministry of Agriculture of Kazakhstan, Document Number: KZ41VCY00098965.

## Author Contributions

NH and JP initiated the LCC study on long-term anesthetized European roe deer and designed as well as conducted the experiment together with FG. PK is the head of the Kulan reintroduction project in Kazakhstan and initiated the evaluation of capture and handling procedures in cooperation with CW and NH. NH conducted all LCC measurements. Data analysis and preparation of figures was done by SV and VM. NH wrote the manuscript with contribution of VM. CW, PK, and VM revised the manuscript. All authors participated in revisions and approved the final manuscript.

### Conflict of Interest Statement

The authors declare that the research was conducted in the absence of any commercial or financial relationships that could be construed as a potential conflict of interest.

## References

[1] Lorimer J Wildlife in the Anthropocene. Conservation After Nature. Minneapolis University of Minnesota Press (2015).

[2] Soulsbury CD White PCL Human–wildlife interactions in urban areas: a review of conflicts, benefits and opportunities. Wildl Res. (2015) 42:541–53. 10.1071/WR14229

[3] Maublanc M-L Bideau E Launay C Monthuir B Gerard J-F Indicators of ecological change (IEC) as efficient tools for managing roe deer populations: a case study. Eur J Wildl Res. (2016) 62:189–97. 10.1007/s10344-016-0992-2

[4] Wikelski M Cooke SJ. Conservation physiology. Trends Ecol Evol. (2006) 21:39–46. 10.1016/j.tree.2005.10.018 16701468

[5] Lynn SE Porter AJ Trapping initiates stress response in breeding and non-breeding house sparrows *Passer domesticus*: implications for using unmonitored traps in field studies. J Avian Biol. (2008) 39:87–94. 10.1111/j.2008.0908-8857.04204.x

[6] Cattet MRL. Falling through the cracks: shortcomings in the collaboration between biologists and veterinarians and their consequences for wildlife. ILAR J. (2013) 54:33–40. 10.1093/ilar/ilt010 23904530

[7] Hampton JO Hyndman TH. Underaddressed animal-welfare issues in conservation. Conserv Biol. (2018) 10.1111/cobi.13267. 30549308

[8] Kirkwood JK. Interventions for wildlife health, conservation and welfare. Vet Rec. (1993) 132:235–8. 10.1136/vr.132.10.235 8460458

[9] Paquet PC Darimont CT Wildlife conservation and animal welfare: two sides of the same coin? Anim Welfare. (2010) 19:177–90.

[10] De la Fuente MF Souto A Caselli C Schiel N People's perception on animal welfare: why does it matter? Ethnobiol Conserv. (2018) 6:18 10.15451/ec2017106.1817

[11] Broom DM. Animal welfare: concepts and measurement. J Anim Sci. (1991) 69:4167–75. 10.2527/1991.69104167x 1778832

[12] American Veterinary Medical Association (AVMA) Animal Welfare: What Is It? [Online]. (2018) Available online at: https://www.avma.org/KB/Resources/Reference/AnimalWelfare/Pages/what-is-animal-welfare.aspx (accessed June 3, 2018).

[13] World Association of Zoos and Aquariums Animal Welfare [Online]. (2018) Available online at: http://www.waza.org/en/site/conservation/animal-welfare-1471340294 (accessed June 3, 2018).

[14] Wiepkema PR Koolhaas JM Stress and animal-welfare. Anim Welfare. (1993) 2:195–218.

[15] Koolhaas JM Korte SM De Boer SF Van Der Vegt BJ Van Reenen CG Hopster H. Coping styles in animals: current status in behavior and stress-physiology. Neurosci Biobehav Rev. (1999) 23:925–35. 10.1016/S0149-7634(99)00026-3 10580307

[16] McEwen BS Wingfield JC. What is in a name? Integrating homeostasis, allostasis and stress. Horm Behav. (2010) 57:105–11. 10.1016/j.yhbeh.2009.09.011 19786032 PMC2815096

[17] Moberg GP Biological response to stress: key to assessment of animal well-being?. In: Moberg GP editors. Animal stress (New York, NY: Springer) (1985). p. 27–49.

[18] Moberg GP Biological response to stress: implications for animal welfare. In: Moberg GP Mench JA editors. The Biology of Animal Stress. Basic Principles and Implications for Animal Welfare (Bethseda Maryland: CABI Publishing) (2000). p. 1–21.

[19] Kirkwood JK Sainsbury AW Bennett PM The welfare of free-living wild animals - methods of assessment. Anim Welfare. (1994) 3:257–73.

[20] McLaren G Bonacic C Rowan A Animal welfare and conservation: measuring stress in the wild. In: Macdonald D Service K editors. Key Topics in Conservation Biology. Oxford: Blackwell (2007). p. 120–33.

[21] Staley M Conners MG Hall K Miller LJ. Linking stress and immunity: Immunoglobulin A as a non-invasive physiological biomarker in animal welfare studies. Horm Behav. (2018) 102:55–68. 10.1016/j.yhbeh.2018.04.011 29705025

[22] Cannon WB Physiological regulation of normal states. Some tentative postulates concerning biological homeostasis. In: Richet ÀC editor. Ses amis, ses collègues, ses élèves. (Paris: Editions Medicales) (1926). p. 91–3.

[23] Selye H A syndrome produced by diverse nocuous agents. Nature. (1936) 138:32–32. 10.1038/138032a0 9722327

[24] Romero LM Dickens MJ Cyr NE. The reactive scope model - a new model integrating homeostasis, allostasis, and stress. Horm Behav. (2009) 55:375–89. 10.1016/j.yhbeh.2008.12.009 19470371

[25] Sterling P Eyer J Allostasis: a new paradigm to explain arousal pathology. In: Fisher K Reason J editors. Handbook of Life Stress, Cognition and Health. Chichester: John Wiley & Sons (1988). p. 629–49.

[26] McEwen BS Wingfield JC. The concept of allostasis in biology and biomedicine. Horm Behav. (2003) 43:2–15. 10.1016/S0018-506X(02)00024-7 12614627

[27] Harvey S Phillips JG Rees A Hall TR. Stress and adrenal function. J Exp Zool. (1984) 232:633–45. 10.1002/jez.1402320332 6097634

[28] Romero LM Wingfield JC Tempests, predators, poxes, and people. Stress in Wild Animals and How They Cope. New York, NY: Oxford University Press (2016).

[29] Sapolsky RM Romero LM Munck AU. How do glucocorticoids influence stress responses? integrating permissive, suppressive, stimulatory, and preparative actions Endocr Rev. (2000) 21:55–89. 10.1210/er.21.1.55 10696570

[30] Korte SM Koolhaas JM Wingfield JC McEwen BS. The Darwinian concept of stress: benefits of allostasis and costs of allostatic load and the trade-offs in health and disease. Neurosci Biobehav Rev. (2005) 29:3–38. 10.1016/j.neubiorev.2004.08.009 15652252

[31] Costantini D Marasco V Møller AP. A meta-analysis of glucocorticoids as modulators of oxidative stress in vertebrates. J Comp Physiol B. (2011) 181:447–56. 10.1007/s00360-011-0566-2 21416253

[32] Martin LB. Stress and immunity in wild vertebrates: timing is everything. Gen Comp Endocrinol. (2009) 163:70–6. 10.1016/j.ygcen.2009.03.008 19318107

[33] McCormick GL Shea K Langkilde T. How do duration, frequency, and intensity of exogenous CORT elevation affect immune outcomes of stress? Gen Comp Endocrinol. (2015) 222:81–7. 10.1016/j.ygcen.2015.07.008 26209864

[34] Marasco V Boner W Heidinger B Griffiths K Monaghan P. Repeated exposure to stressful conditions can have beneficial effects on survival. Exp Gerontol. (2015) 69:170–5. 10.1016/j.exger.2015.06.011 26093051

[35] Marasco V Boner W Griffiths K Heidinger B Monaghan P. Environmental conditions shape the temporal pattern of investment in reproduction and survival. Proc R Soc Lond B. (2018) 285:20172442. 10.1098/rspb.2017.2442 29298939 PMC5784202

[36] Castle K Gillin C Hernandez S Justice-Allen A Lamberski N Nichols M. Preface to and acknowledgments for the journal of wildlife diseases special supplement: advances and improvements in wildlife welfare. J Wildl Dis. (2016) 52:S1–3. 10.7589/52.2s.S1 26845293

[37] Journal of Wildlife Diseases Wildlife welfare supplement editorial board. adv animal welfare free-living animals. J Wildl. Dis. (2016) 52:S4–13. 10.7589/52.2s.S4 26845298

[38] Romero LM Remage-Healey L. Daily and seasonal variation in response to stress in captive starlings (*Sturnus vulgaris*): corticosterone. Gen Comp Endocrinol. (2000) 119:52–9. 10.1006/gcen.2000.7491 10882549

[39] Koolhaas JM de Boer SF Buwalda B van Reenen K. Individual variation in coping with stress: a multidimensional approach of ultimate and proximate mechanisms. Brain Behav. Evol. (2007) 70:218–26. 10.1159/000105485 17914253

[40] Cockrem JF. Individual variation in glucocorticoid stress responses in animals. Gen Comp Endocrinol. (2013) 181:45–58. 10.1016/j.ygcen.2012.11.025 23298571

[41] Nelson RJ Demas GE Klein SL Kriegsfeld LJ Seasonal Patterns of Stress, Immune Function, and Disease. New York, NY: Cambridge University Press (2002).

[42] Barrio IC Hik DS Bueno CG Cahill JF Extending the stress-gradient hypothesis - is competition among animals less common in harsh environments? Oikos. (2013) 122:516–23. 10.1111/j.1600-0706.2012.00355.x

[43] Romero LM Platts SH Schoech SJ Wada H Crespi E Martin LB Understanding stress in the healthy animal – potential paths for progress. Stress. (2015) 15:491–7. 10.3109/10253890.2015.1073255 26365223

[44] Möstl E Palme R. Hormones as indicators of stress. Domest Anim Endocr. (2002) 23:67–74. 10.1016/S0739-7240(02)00146-7 12142227

[45] Macbeth BJ Cattet MRL Stenhouse GB Gibeau ML Janz DM Hair cortisol concentration as a noninvasive measure of long-term stress in free-ranging grizzly bears (*Ursus arctos*): considerations with implications for other wildlife. Can J Zool. (2010) 88:935–49. 10.1139/Z10-057

[46] Yamanashi Y Is hair cortisol useful for animal welfare assessment? review of studies in captive chimpanzees. Aquat Mamm. (2018) 44:201–10. 10.1578/AM.44.2.2018.201

[47] Davis AK Maney DL Maerz JC The use of leukocyte profiles to measure stress in vertebrates: a review for ecologists. Funct Ecol. (2008) 22:760–72. 10.1111/j.1365-2435.2008.01467.x

[48] Rushen J Some issues in the interpretation of behavioural responses to stress. In: MG Mench J editors. The Biology of Animal Stress. Basic principles and implications for animal welfare (Wallingford, UK: CAB International) (2000). p. 23–42.

[49] Koolhaas JM Bartolomucci A Buwalda B de Boer SF Flügge G Korte SM. Stress revisited: A critical evaluation of the stress concept. Neurosci Biobehav Rev. (2011) 35:1291–301. 10.1016/j.neubiorev.2011.02.003 21316391

[50] Otovic P Hutchinson E. Limits to using HPA axis activity as an indication of animal welfare. Altex. (2015) 32:41–50. 10.14573/altex.1406161 25418851

[51] Huber N Fusani L Ferretti A Mahr K Canoine V. Measuring short-term stress in birds: Comparing different endpoints of the endocrine-immune interface. Physiol Behav. (2017) 182:46–53. 10.1016/j.physbeh.2017.09.017 28958953

[52] Taub DD. Neuroendocrine interactions in the immune system. Cell Immunol. (2008) 252:1–6. 10.1016/j.cellimm.2008.05.006 18619587 PMC2562609

[53] Deak T Quinn M Cidlowski JA Victoria NC Murphy AZ Sheridan JF. Neuroimmune mechanisms of stress: sex differences, developmental plasticity, and implications for pharmacotherapy of stress-related disease. Stress. (2015) 18:367–80. 10.3109/10253890.2015.1053451 26176590 PMC4813310

[54] Verburg-van Kemenade BML Cohen N Chadzinska M. Neuroendocrine-immune interaction: evolutionarily conserved mechanisms that maintain allostasis in an ever-changing environment. Dev Comp Immunol. (2017) 66:2–23. 10.1016/j.dci.2016.05.015 27296493

[55] Ader R. On the development of psychoneuroimmunology. Eur J Pharmacol. (2000) 405:167–76. 10.1016/S0014-2999(00)00550-1 11033324

[56] Adamo SA. The effects of the stress response on immune function in invertebrates: an evolutionary perspective on an ancient connection. Horm Behav. (2012) 62:324–30. 10.1016/j.yhbeh.2012.02.012 22381405

[57] Swan MP Hickman DL. Evaluation of the neutrophil-lymphocyte ratio as a measure of distress in rats. Lab Anim. (2014) 43:276. 10.1038/laban.529 25050728

[58] Davis AK Maney DL The use of glucocorticoid hormones or leucocyte profiles to measure stress in vertebrates: what's the difference? Methods Ecol. Evol. (2018) 9:1556–1568. 10.1111/2041-210x.13020

[59] Singh A Immunology as applied to farm animal welfare: general principles and interventions. RRJol. (2018) 8:1–13.

[60] Breineková K Svoboda M Smutná M Vorlová L. Markers of acute stress in pigs. Physiol Res. (2007) 56:323–9. 16792472 10.33549/physiolres.930938

[61] Mantovani A Cassatella MA Costantini C Jaillon S. Neutrophils in the activation and regulation of innate and adaptive immunity. Nat Rev Immunol. (2011) 11:519–31. 10.1038/nri3024 21785456

[62] Huff GR Dutta V Huff WE Rath NC. Effects of dietary yeast extract on turkey stress response and heterophil oxidative burst activity. Br Poultry Sci. (2011) 52:446–55. 10.1080/00071668.2011.600753 21919572

[63] Ortega E Giraldo E Hinchado MD Martín L García JJ De la Fuente M. Neuroimmunomodulation during Exercise: role of catecholamines as ‘stress mediator' and/or ‘danger signal' for the innate immune response. NeuroImmunoModulation. (2007) 14:206–12. 10.1159/000110648 18073516

[64] Brown AS Levine JD Green PG. Sexual dimorphism in the effect of sound stress on neutrophil function. J Neuroimmunol. (2008) 205:25–31. 10.1016/j.jneuroim.2008.08.005 18838177

[65] Schleimer RP Freeland HS Peters SP Brown KE Derse CP. An assessment of the effects of glucocorticoids on degranulation, chemotaxis, binding to vascular endothelium and formation of leukotriene B_4_ by purified human neutrophils. J Pharmacol Exp Ther. (1989) 250:598–605. 2547940

[66] Robinson JM. Phagocytic leukocytes and reactive oxygen species. Histochem Cell Biol. (2009) 131:465–9. 10.1007/s00418-009-0565-5 19224236

[67] Hultqvist M Olofsson P Holmberg J Bäckström BT Tordsson J Holmdahl R. Enhanced autoimmunity, arthritis, and encephalomyelitis in mice with a reduced oxidative burst due to a mutation in the *Ncf1* gene. Proc Natl Acad Sci USA. (2004) 101:12646–51. 10.1073/pnas.0403831101 15310853 PMC515111

[68] Bennett N Maglione PJ Wright BL Zerbe C. Infectious complications in patients with chronic granulomatous disease. J Pediatric Infect Dis Soc. (2018) 7:S12–7. 10.1093/jpids/piy013 29746678 PMC5985728

[69] Viswanathan K Daugherty C Dhabhar FS. Stress as an endogenous adjuvant: augmentation of the immunization phase of cell-mediated immunity. Int Immunol. (2005) 17:1059–69. 10.1093/intimm/dxh286 16000327

[70] Dhabhar FS. Enhancing versus suppressive effects of stress on immune function: implications for immunoprotection and immunopathology. NeuroImmunoModulation. (2009) 16:300–17. 10.1159/000216188 19571591 PMC2790771

[71] Råberg L Grahn M Hasselquist D Svensson E. On the adaptive significance of stress-induced immunosuppression. Proc R Soc B. (1998) 265:1637–41. 10.1098/rspb.1998.0482. 9753786 PMC1689346

[72] Mian R McLaren G Macdonald DW Of stress, mice and men: a radical approach to old problems in stress and health. In: Oxington KV editors. Stress and Health: New Research. (New York, NY: Nova Sience Publishers) (2005). p. 61–79.

[73] Biondi M Effects of stress on immune function: an overview. In: Ader R Felten DL Cohen N editors. Psychoneuroimmunology (San Diego, CA: Academic Press) (2001). p. 189–226.

[74] Dhabhar FS. Effects of stress on immune function: the good, the bad, and the beautiful. Immunol Res. (2014) 58:193–210. 10.1007/s12026-014-8517-0 24798553

[75] Hegemann A Marra PP Tieleman BI. Causes and consequences of partial migration in a passerine bird. Am Nat. (2015) 186:531–46. 10.1086/682667 26655576

[76] McLaren GW Macdonald DW Georgiou C Mathews F Newman C Mian R. Leukocyte coping capacity: a novel technique for measuring the stress response in vertebrates. Exp Physiol. (2003) 88:541–6. 10.1113/Eph8802571 12861342

[77] Shelton-Rayner GK Mian R Chandler S Robertson D Macdonald DW Leukocyte responsiveness, a quantitative assay for subjective mental workload. Int J Ind Ergonom. (2012) 42:25–33. 10.1016/j.ergon.2011.11.004

[78] Thompson I White A Fletcher TC Houlihan DF Secombes CJ The effect of stress on the immune response of Atlantic salmon (*Salmo salar* L.) fed diets containing different amounts of vitamin C. Aquaculture. (1993) 114:1–18. 10.1016/0044-8486(93)90246-U

[79] Montes I McLaren GW Macdonald DW Mian R The effect of transport stress on neutrophil activation in wild badgers (*Meles meles*). Anim Welfare. (2004) 13:355–9.

[80] Mian R MacDonald DW Determining Coping Capacity After Exposure to a Psychological Stressor. United States Patent US 7,838,262 B2, 435/25. 10/533,935. C12Q 1/26 (2006.01) (2010).

[81] Lilius EM Marnila P. Photon emission of phagocytes in relation to stress and disease. Experientia. (1992) 48:1082–91. 1473571 10.1007/BF01947995

[82] Graham SP Kelehear C Brown GP Shine R. Corticosterone-immune interactions during captive stress in invading Australian cane toads (*Rhinella marina*). Horm Behav. (2012) 62:146–53. 10.1016/j.yhbeh.2012.06.001 22713726

[83] Esteruelas NF Huber N Evans AL Zedrosser A Cattet M Palomares F. Leukocyte coping capacity as a tool to assess capture- and handling-induced stress in scandinavian brown bears (*Ursus arctos*). J Wildl Dis. (2016) 52:S40–53. 10.7589/52.2S.S40 26845299

[84] Huber N Vetter SG Evans AL Kjellander P Küker S Bergvall UA. Quantifying capture stress in free ranging European roe deer (*Capreolus capreolus*). BMC Vet Res. (2017) 13:127. 10.1186/s12917-017-1045-0 28490331 PMC5424289

[85] El-Benna J Hurtado-Nedelec M Marzaioli V Marie JC Gougerot-Pocidalo MA Dang PMC. Priming of the neutrophil respiratory burst: role in host defense and inflammation. Immunol Rev. (2016) 273:180–93. 10.1111/imr.12447 27558335

[86] Winters KRH Meyer E Van Merris VM Van Den Broeck WLM Duchateau L Burvenich C Sex steroid hormones do not influence the oxidative burst activity of polymorphonuclear leukocytes from ovariectomized cows *in vitro*. Steroids. (2003) 68:397–406. 10.1016/S0039-128X(03)00040-0 12798490

[87] Oertelt-Prigione S. The influence of sex and gender on the immune response. Autoimmun Rev. (2012) 11:A479–85. 10.1016/j.autrev.2011.11.022 22155201

[88] Sato S Takahashi I Komame H Akimoto N Sawada K Tanaka R Association of sex steroid hormones with neutrophil function in the general population. Hirosaki Med. J. (2016) 67:13–27.

[89] Smulders D Verbeke G Mormède P Geers R. Validation of a behavioral observation tool to assess pig welfare. Physiol Behav. (2006) 89:438–47. 10.1016/j.physbeh.2006.07.002 16904137

[90] Carroll GA Boyle LA Hanlon A Palmer MA Collins L Griffin K. Identifying physiological measures of lifetime welfare status in pigs: exploring the usefulness of haptoglobin, C- reactive protein and hair cortisol sampled at the time of slaughter. Irish Vet J. (2018) 71:8. 10.1186/s13620-018-0118-0 29507716 PMC5833096

[91] Serra M Wolkers CP Urbinati EC Physiological indicators of animal welfare. Revista Brasileira de Zoociências. (2018) 19:70–96.

[92] Tilbrook AJ Ralph CR Hormones, stress and the welfare of animals. Anim Prod Sci. (2018) 58:408–15. 10.1071/AN16808

[93] Arnemo JM Ahlqvist P Andersen R Berntsen F Ericsson G Odden J Risk of capture-related mortality in large free-ranging mammals: experiences from Scandinavia. Wildl Biol. (2006) 12:109–13. 10.2981/0909-6396(2006)12[109:Rocmil]2.0.Co;2

[94] Barros DSB Evans AL Arnemo JM Stenbacka F Ericsson G. Effective thiafentanil immobilization and physiological responses of free-ranging moose (*Alces alces*) in northern Sweden. Vet Anaesth Analg. (2018) 45:502–9. 10.1016/j.vaa.2018.02.008 29891211

[95] Nishina K Akamatsu H Mikawa K Shiga M Maekawa N Obara H. The inhibitory effects of thiopental, midazolam, and ketamine on human neutrophil functions. Anesth Analg. (1998) 86:159–65. 10.1097/00000539-199801000-00032 9428872

[96] Kiefer R-T Ploppa A Krueger Wolfgang A Plank M Nohé B Haeberle Helene A. Local anesthetics impair human granulocyte phagocytosis activity, oxidative burst, and cd11b expression in response to *Staphylococcus aureus*. Anesthesiology. (2003) 98:842–848. 12657844 10.1097/00000542-200304000-00009

[97] Kurosawa S Kato M. Anesthetics, immune cells, and immune responses. J Anesth. (2008) 22:263–77. 10.1007/s00540-008-0626-2 18685933

[98] Fröhlich D Rothe G Schwall B Schmid P Schmitz G Taeger K. Effects of volatile anaesthetics on human neutrophil oxidative response to the bacterial peptide FMLP. Brit J Anaesth. (1997) 78:718–23. 10.1093/bja/78.6.718 9215026

[99] Billert H Czerniak K Bednarek E Kulinska K. Effects of local anesthetics on the respiratory burst of cord blood neutrophils *in vitro*. Pediatr Res. (2016) 80:258–66. 10.1038/pr.2016.68 27055189

[100] Palić D Herolt DM Andreasen CB Menzel BW Roth JA Anesthetic efficacy of tricaine methanesulfonate, metomidate and eugenol: effects on plasma cortisol concentration and neutrophil function in fathead minnows (*Pimephales promelas* Rafinesque, 1820). Aquaculture. (2006) 254, 675–685. 10.1016/j.aquaculture.2005.11.004

[101] Gelling M Montes I Moorhouse TP Macdonald DW. Captive housing during water vole (*Arvicola terrestris*) reintroduction: does short-term social stress impact on Animal welfare? PLoS ONE. (2010) 5:e9791. 10.1371/journal.pone.0009791. 20352093 PMC2844416

[102] Gelling M McLaren GW Mathews F Mian R Macdonald DW Impact of trapping and handling on leukocyte coping capacity in bank votes (*Clethrionomys glareolus*) and wood mice (*Apodemus sylvaticus*). Anim Welfare. (2009) 18:1–7.

[103] Moorhouse TP Gelling M McLaren GW Mian R Macdonald DW Physiological consequences of captive conditions in water voles (*Arvicola terrestris*). J Zool. (2007) 271:19–26. 10.1111/j.1469-7998.2006.00175.x

[104] Honess PE Marin C Brown AP Wolfensohn SE Assessment of stress in non-human primates: application of the neutrophil activation test. Anim Welfare. (2005) 14:291–5.

[105] Gaudio E Bordin S Lora I Lora M Massignani M De Benedictis GM. Leukocyte coping capacity chemiluminescence as an innovative tool for stress and pain assessment in calves undergoing ring castration. J Anim Sci. (2018) 96:4579–89. 10.1093/jas/sky342 30137392 PMC6247858

[106] Wingfield JC Ramenofsky M Hormones and the behavioral ecology of stress. In: Stress physiology in animals. ed PHM Balm (Sheffield: Sheffield Academic Press) (1999). p. 1–51.

[107] Kaczensky P Linnell JDC Zuther S Salemgareyev AR Doldi R Reintroduction of kulan into the central steppe of Kazakhstan: Field Report for 2017. NINA Report. (2018). Available online at: www.nina.no

[108] Fraser D Assessing animal well-being: common sense, uncommon science. In: ed. Purdue University; Office of Agricultural Research Programs Food animal well-being. (Purdue University) (1993). p. 37–54.

