# Supplementary material for: Leukocyte Coping Capacity: An Integrative Parameter for Wildlife Welfare Within Conservation Interventions
Source: Front Vet Sci. 2019 Apr 11;6:105. doi: 10.3389/fvets.2019.00105 (PMC6470256; doi:10.3389/fvets.2019.00105)
Supplement: Supplementary file 1 [file Data_Sheet_1.pdf]

Supplementary Material

S1: LCC protocol

To measure unstimulated blood chemiluminescence levels, providing information on the individual baseline level of reactive oxygen species (ROS), 10 µl of lithium heparinized whole blood were transferred into a silicon antireflective tube (Lumivial, EG & G Berthold, Germany). We added 90 µl of 10^{-4} mol l^{-1} luminol (5-amino-2,3-dihydrophthalazine-1,4-dione; VWR International, Stockholm, Sweden) dissolved in dimethyl sulfoxide (DMSO; Sigma-Aldrich (now Merck), Darmstadt, Germany) and diluted with phosphate-buffered saline (PBS, pH 7.4). Subsequently 10 µl of PBS were added and the tube was shaken gently for mixing. Luminol produces chemiluminescence when combined with an oxidizing agent (i.e. ROS), producing a low-intensity light reaction (Merényi et al., 1990). To measure full blood chemiluminescence produced in response to a secondary challenge (the first challenge is the stress reaction in vivo), a second tube was prepared in parallel as described above but 10 µl of 10^{-5} mol l^{-1} phorbol 12-myristate 13-acetate (PMA; Sigma-Aldrich (now Merck), Darmstadt, Germany) was added instead of 10 µl PBS. The higher the extent of the first challenge (i.e., the stress reaction of the animal), the lower the chemiluminescence response to the chemical secondary challenge is going to be. In other words, low LCC values indicate high stress levels and vice versa.

Blood chemiluminescence for each tube was assessed every 5 min for a total of 30 s over a period of 30 min and expressed in relative light units (RLU), using a portable high sensitivity chemiluminometer (Junior LB 9509, EG & G Berthold, Germany). All measurements were carried out in a closed environment (kulan project - inside a car; roe deer project - in the surgery room), ensuring stable conditions above 15°C and were performed immediately after the blood sample was collected. When not in the chemiluminometer, tubes were incubated at 37°C in a lightproof metal bead bath (Minitüb, Tiefenbach, Germany). In order to correct for background noise, we subtracted the values of the control sample from that of the challenged sample measured at the same timepoint. From the resulting LCC response curve we extracted the LCC peak as variable. LCC peak performance was defined as the maximum ROS production of PMNL´s in the course of the oxidative burst (see Figure S1).

S2: Methodology and more detailed results from the two ongoing wildlife projects

Project descriptions

For detailed information on the ongoing translocation project of kulan in Kazakhstan, the species as well as the involved procedures see (Kaczensky et al., 2017; Kaczensky et al., 2018); https://www.nina.no/english/Research/KULANSTEP). In brief, kulan had been driven into a capture corral, rested overnight and then anaesthetised via remote darting and subsequently sampled, radio-collared, and boxed for translocation the following day (Kaczensky et al., 2018). The Kulan project (ecological assessment) was approved by the Committee of Forestry and Wildlife (CFW) of the Ministry of Agriculture of Kazakhstan, Document Number: KZ41VCY00098965".

In the ongoing roe deer project 9 European Roe deer (*Capreolus capreolus*) males were kept at the Leibniz-Institute for Zoo- and Wildlife Research, Niederfinow, Germany (52°44'N, 13°50'E). Experiments were conducted in the end of January and mid of June 2015 (winter 2015 and summer 2015, respectively), as well as beginning of February 2016 (winter 2016) over four consecutive days in every season. Roe deer bucks within the study are part of a captive population at the field station and are held in groups between 3-5 individuals in 1000-2000 m^2 outdoor enclosures. The animals were driven one by one into a small stand and anaesthetized (either Xylazine 4 mg/kg^{-1} in 2015 (Rompun, Bayer, Germany) or Medetomidine 0.05 mg/kg^{-1} in 2016 (Domitor, Orion, Espoo, Finland), and Ketamine 4 $mg/\ kg^{-1}$ (Ketamin, Albrecht, Germany)), via i.m. injection using a blow pipe. A first jugular blood sample for LCC analysis was taken immediately after recumbency (0 min). Subsequently the animal was transported for 2 min to a surgery room at the research station. The animals were intubated, received 2L min^{-1} oxygen and 15 mL/kg/hr isotonic infusion. Further blood samples were taken at 40, 80 and 120 min after the first blood sample. At 120 min the animals were given 0.1mg kg^{-1} Atipamezol (Antisedan, Orion, Espoo, Finnland) for antagonisation and transported back into their enclosure for recovery from anesthesia.

All experimental procedures were approved by the German Ministry for Environment, Health and consumer protection (AZ: 2347-4-2015).

In both projects LCC measurements were carried out according to the standard protocol described in Supplementary Material S1.

Statistical analysis and results

Using the LCC data of Kulan a linear double logarithmic model was calculated in order to test whether LCC peak values (the highest increase of ROS production within the 30 minute measurement period) were representative of the whole LCC curve (i.e. area under the curve; AUC). This model confirmed that LCC peak values are a robust proxy for the entire LCC curve measured (slope ± s.e. = 0.93 ± 0.08, $t = 11.58$, $p < 0.001$, adj. $R^2 = 0.92$; Figure 2 in the main manuscript). Comparing LCC peak values of the two prematurely released individuals versus the 10 transported kulan in a linear

model, we found the latter to have significantly higher LCC levels (estimated difference ± s.e. = 767.4 ± 255.2, t = 3.01, p = 0.013, adj. $R^2 = 0.42$; Figure 1 in the main manuscript).

We used General Linear Mixed Models to analyse whether LCC values of roe deer during the 120 min period of anesthesia varied with sampling time, across seasons, with age and/or in dependence of the handling time prior to anesthesia (R packages used: "lme4" (Bates et al., 2015) and "lmerTest" (Kuznetsova et al., 2016)). Additionally, we included the interaction of the sampling time and season in the model, as well as the handling time prior to anesthesia and the year of birth of the animals. Individual ID was included as random factor to account for repeated measurements. After performing a backward model selection based on likelihood ratio test we found that season and sampling time significantly affected LCC levels in roe deer independently (F = 25.32, $p < 0.001$ and F = 4.10, $p = 0.010$, respectively) but not their interaction, the age of the animals or the handling time prior to anesthesia (all $p \geq 0.34$). LCC values during summer were markedly higher compared to two winter seasons ($p < 0.001$ for both comparisons; R package used: "lsmeans" (Lenth, 2016); Figure 3 in the main manuscript). We further identified significant differences in LCC levels over sampling time (F = 4.10, $p = 0.010$). Although *post-hoc* tests revealed a significant difference only between t = 0 min (T0) and t = 80 min (T80; $p = 0.010$; all other $p \geq 0.11$; R package used: "lsmeans" (Lenth, 2016)), the data suggested a gradual recovery of LCC during anesthesia (Figure 4 in the main manuscript).

All analyses on the two preliminary data sets on kulan and European roe deer LCC were performed in R (version 3.5.0; R Core Team (2015)). None of the models showed serious deviations from normality according to the graphical inspection of residuals via histograms and quantile-quantile plots or signs for heteroscedasticity.

Supplementary Figure S1

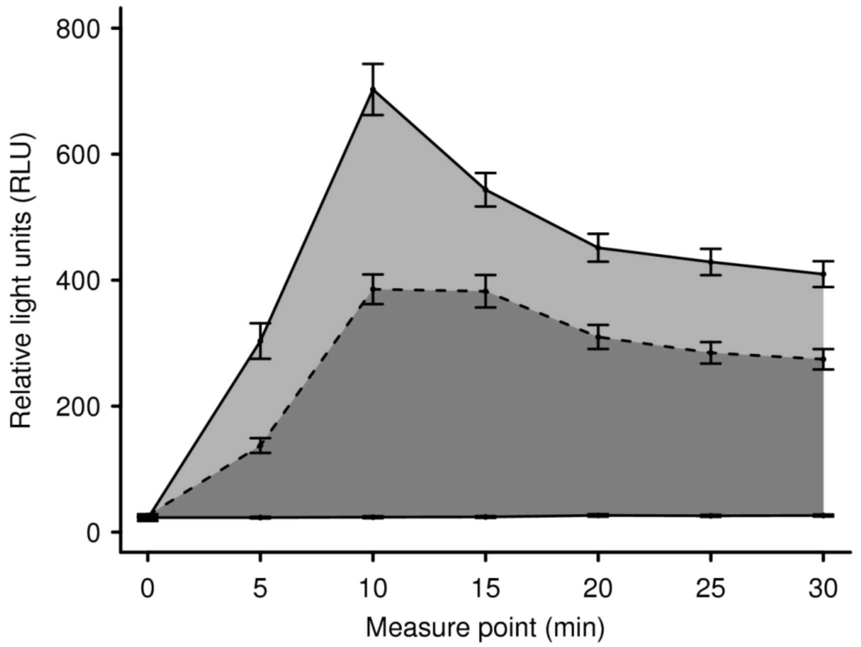

Figure S1. Leukocyte Coping Capacity (LCC) curves (measured every 5 min for 30 s over 30 min) for anaesthetized European roe deer males (n = 9). Blood samples from all animals were taken in the seasons of winter 2015, summer 2015 and winter 2016. The lower line represents basal levels of reactive oxygen species (ROS) whereas the upper lines represent PMA stimulated samples. The dark grey shaded area indicates the integral of the area under the curve (AUC) representing the LCC response in the winter seasons of 2015 and 2016. The entire gray area represents the AUC of the LCC response in summer 2016. Data points represent mean LCC levels (in relative light units) of blood samples taken immediately after recumbency, 40, 80, and 120 min after the first sample, with error bars showing the standard error of the mean for each time point (not visible in the baseline due to very low variance).

Bates, D., Mächler, M., Bolker, B., and Walker, S. (2015). Fitting Linear Mixed-Effects Models Using lme4. *J Stat. Softw.* 67(1), 1-48. doi: 10.18637/jss.v067.i01.

Kaczensky, P., Doldin, R., Enke, D., Linnell, J.D., Lukanovsky, O., Salemgareyev, A.R., et al. (2017). *Feasibility study for kulan (Equus hemionus kulan) reintroduction into the central steppe of Kazakhstan.* NINA Report.

Kaczensky, P., Linnell, J.D.C., Zuther, S., Salemgareyev, A.R., and Doldi, R. (2018). *Reintroduction of kulan into the central steppe of Kazakhstan: Field Report for 2017.* NINA Report.

Kuznetsova, A., Brockhoff, P.B., and Christensen, R.H.B. (2016). *Tests in Linear Mixed Effects Models Version 2.0-30* [Online]. Available: http://CRAN.R-project.org/package=lmerTest [Accessed].

Lenth, R.V. (2016). Least-Squares Means: The R Package lsmeans. *J Stat. Softw.* 69(1), 1-33. doi: 10.18637/jss.v069.i01.

Merényi, G., Lind, J., and Eriksen, T.E. (1990). Luminol Chemiluminescence: Chemistry, Excitation, Emitter. *J Biolumin. Chemilumin.* 5(1), 53-56. doi: 10.1002/bio.1170050111.

R Core Team (2015). *R: A language and environment for statistical computing. R Foundation for Statistical Computing, Vienna, Austria* [Online]. Available: http://www.R-project.org/ [Accessed].
